# Comparing specificity and seroconversion sensitivity among major blood screening assays for human immunodeficiency virus and viral hepatitis

**DOI:** 10.1111/vox.70222

**Published:** 2026-02-17

**Authors:** Monica Chaves, Martina Schaetzl, Florina Pessl

**Affiliations:** ^1^ Roche Diagnostics International Rotkreuz Switzerland; ^2^ Roche Diagnostics GmbH Penzberg Germany

**Keywords:** blood screening, human immunodeficiency virus, immunoassays, seroconversion sensitivity, specificity, viral hepatitis

## Abstract

**Background and Objectives:**

Accurate detection of transfusion‐transmissible infections, such as human immunodeficiency virus (HIV) and hepatitis C and B viruses (HCV/HBV), is critical to ensure blood safety, and screening assays must demonstrate high specificity and sensitivity. In this study, we compared the performances of Elecsys® HIV Duo, HCV Duo, Anti‐HCV II and HBsAg II immunoassays with those of comparator assays HIV Ag/Ab Combo, Anti‐HCV II and HBsAg on the Alinity® s platform and HIV Ag/Ab Combo, Anti‐HCV and HBsAg Qualitative II on the Alinity i platform.

**Materials and Methods:**

Approximately 2050 plasma samples from first‐time blood donors and commercial seroconversion panels were used. Specificity was assessed as the proportion of true non‐reactive samples identified by each assay. Seroconversion sensitivity was evaluated based on the detection time and average interval from nucleic acid testing (NAT)‐positivity to assay reactivity.

**Results:**

Overall, all Elecsys assays evaluated had similar specificities as the corresponding Alinity s/i assays, although the absolute difference in specificity between Alinity i Anti‐HCV assay and the other HCV assays was statistically significant. The seroconversion sensitivities of Elecsys HIV Duo and HBsAg II assays were similar to the corresponding Alinity s/i assays. For HCV, Elecsys HCV Duo assay detected infection earlier than Alinity s/i assays for most panels (87.0% and 91.8%, respectively), with an average detection time from NAT positivity for HCV RNA of 1.5 days versus 21.4 and 23.8 days, respectively.

**Conclusion:**

The robust specificities and early detection capabilities of the evaluated Elecsys assays support their routine use in blood donor screening and diagnosis.

**Highlights**

- The large number of seroconversion panels and samples from first‐time blood donors used in this study minimized preselection bias and enabled comprehensive comparison of early detection capabilities, thereby validating the performance of the Elecsys® HIV Duo, HCV Duo, Anti‐HCV II and HBsAg II immunoassays in real‐world blood screening scenarios.
- The considerably shorter average time interval between assay reactivity and nucleic acid testing (NAT) positivity for the Elecsys HCV Duo assay (1.5 days) could narrow the serological window and enhance early diagnosis, especially in settings without routine NAT.
- The high specificity and early detection capabilities of the Elecsys HIV Duo, HCV Duo, Anti‐HCV II and HBsAg II immunoassays bolster their suitability for routine blood‐donor screening and diagnosis of transfusion‐transmissible infections, contributing to improved blood safety and public health outcomes.

## INTRODUCTION

According to the World Health Organization's (WHO) global health sector strategies for 2022–2030, strategically focused responses must be implemented to eradicate human immunodeficiency virus (HIV) infection, viral hepatitis and sexually transmitted or transfusion‐transmitted infections [1]. Most countries are not on track to meet the WHO's targets for reasons including gaps in diagnostic access, treatment delivery, efficient preventative measures and linkage to care [2, 3]. HIV continues to be a major global health challenge, with over 1 million new infections annually, and timely diagnosis through frequent testing is essential to ensure early access to care and treatment [4]. Similarly, the burden of hepatitis B virus (HBV) remains high—the WHO estimated that in 2022, 254 million people were living with chronic HBV infection globally, with 1.1 million associated deaths [5]. Globally, hepatitis C virus (HCV) contributes significantly to morbidity and mortality, with an estimated 50 million people living with chronic hepatitis C [6].

Guidelines recommend universal testing in regions with high HIV burden across all populations and healthcare settings [7]. The United States Centers for Disease Control and Prevention (CDC) recommends HCV screening at least once in a lifetime for adults aged 18 years or older and during all pregnancies, except in settings where the infection prevalence is <0.1%. Additionally, it stipulates that, regardless of age or prevalence, individuals with risk factors should be tested for HCV, with intermittent testing while risk factors persist [8]. In settings where the general population has intermediate to high HCV antibody (Ab) seroprevalence (i.e., ≥2%), the WHO recommends routine HCV testing for all adolescents and adults, irrespective of individual risk factors [6]. For HBV, the WHO and CDC advise periodic testing of the most affected populations, such as those with high HBV seroprevalence or history of exposure, regardless of age [5, 9]. In settings with intermediate (≥2%) or high (≥5%) hepatitis B surface antigen (HBsAg) seroprevalence in the general population, all adults should undergo routine HBsAg serological testing [5]. Facility‐ and community‐based hepatitis testing services are advised to adopt strategies to enhance testing uptake and ensure linkage to care [5, 10].

As part of the strategic response for elimination of transfusion‐transmissible infectious diseases, the WHO mandates comprehensive screening of blood products [1, 5]; screening techniques must have high specificity and sensitivity in order to ensure the highest standards of blood safety [11]. Immunoassays have evolved from basic screening tools to sophisticated platforms for clinical diagnostics, blood screening and epidemiological surveillance [12, 13]. Automated immunoassay analysers such as the Cobas® e 801 system (e 801 analyser; Roche Diagnostics International, Rotkreuz, Switzerland), Alinity® s (Abbott GmbH & Co. KG, Wiesbaden, Germany) and Alinity i (Abbott GmbH & Co. KG) enable high‐throughput processing of serological immunoassays for accurate and rapid detection of biomarkers in human serum and plasma [14]. The Abbott Alinity s and Alinity i platforms process chemiluminescent microparticle immunoassays to detect various viruses, including HIV, HBV and HCV [15, 16].

Elecsys® is a comprehensive series of electrochemiluminescence technology–based immunoassays designed for use on Cobas e analysers. The Elecsys HIV Duo immunoassay facilitates in vitro qualitative evaluation of HIV‐1 p24 antigen and antibodies to HIV‐1 and HIV‐2 in human serum and plasma. Similarly, the Elecsys HCV Duo assay detects HCV core antigen and antibodies to HCV. For both immunoassays, antigen and antibody sub‐results guide the selection of confirmatory algorithms for reactive samples. The Elecsys Anti‐HCV II immunoassay detects antibodies to HCV in human serum and plasma, while the Elecsys HBsAg II immunoassay is designed to detect the large polypeptide HBsAg, an important component of the HBV envelope.

The present study aimed to evaluate performances of the Elecsys HIV Duo, Elecsys Anti‐HCV II, Elecsys HCV Duo and Elecsys HBsAg II assays. We compared the specificities and seroconversion sensitivities of these assays on the e 801 analyser with those of HIV Ag/Ab Combo, Anti‐HCV II and HBsAg assays on the Alinity s platform and HIV Ag/Ab Combo, Anti‐HCV and HBsAg Qualitative II assays on the Alinity i platform.

## MATERIALS AND METHODS

This was a comparative analysis of the specificities and seroconversion sensitivities of Elecsys HIV Duo, Anti‐HCV II, HCV Duo and HBsAg II immunoassays on the e 801 analyser against HIV Ag/Ab Combo, Anti‐HCV II and HBsAg immunoassays on the Alinity s platform and HIV Ag/Ab Combo, Anti‐HCV and HBsAg Qualitative II immunoassays on the Alinity i platform.

Assay specificity was determined using 2048 pseudonymized, fresh (no freeze/thaw cycles) plasma samples from first‐time blood donors (2046 for HCV). Samples were tested under the same conditions for all three systems. If the sample was initially reactive (IR) or repeatedly reactive (RR) on any platform, confirmatory testing resolution was performed (Supplementary Methods). No additional blood draws or follow‐up samples were collected.

Seroconversion sensitivity was evaluated following a descriptive, comparative approach using 183 commercial seroconversion panels (HIV, *n* = 51; HCV, *n* = 71; HBV, *n* = 61) provided by Roche, which represent sequential specimens collected from individuals during the early phase of infection, allowing for longitudinal analysis of seroconversion dynamics. For each assay, the proportion of seroconversion panels for which the assay yielded at least one reactive result was determined; further, the earliest time point (i.e., sample draw) at which infection was detected was identified. As a quantitative metric, the average interval from the first nucleic acid testing (NAT)‐positive result (identified from the seroconversion panel package inserts; defined as Day 0) to the first reactive result for each immunoassay was estimated. This was done for a subset of panels, because for some panels the package inserts did not report any positive results by molecular testing, or all draws yielded negative results with all assays. Samples were tested under the same conditions for all three systems.

For the Alinity platforms, seroconversion panels were tested externally at Sanquin Blood Supply Foundation (Amsterdam, the Netherlands), while Elecsys immunoassays were tested internally at Roche Diagnostics, Research & Development, Penzberg, Germany. Final seroconversion sensitivity results combined data from both testing locations.

### Data analysis

Results were interpreted according to the manufacturers' package inserts (Tables S1–S3).

Specificity calculations were based on the confirmatory algorithm (Supplementary Methods), which was used consistently across all assays, to establish the final confirmed status of each sample. Specificity—expressed as the percentage of true non‐reactive samples correctly identified by each assay—was calculated alongside corresponding 95% confidence intervals (CIs). Statistical significance was determined based on the limits of the two‐sided CIs. Differences with lower CI limits exceeding 0 were deemed statistically significant [17].

The formula used to calculate specificity was as follows:

$$\text{Specificity}\;\left(\%\right)=D/\left(B+D\right)\times 100,$$

where *B* is the number of false positives, and *D* is the number of true negatives.

For the seroconversion sensitivity analysis, seroconversion panel package inserts were systematically reviewed to determine the timing of the first NAT‐positive result for each panel. This was used as the point of infection onset (Day 0).

### Ethics statement

The study protocol and its amendments were approved by the institutional review board of the Sanquin Blood Supply Foundation. Informed consent was not required because pseudonymized remnant specimens were used.

## RESULTS

### Specificity

Table 1 shows the specificity results; all evaluated assays met the minimum specificity threshold of ≥99.5% [18]. For HIV, 2048 samples were tested. The Elecsys HIV Duo assay demonstrated 99.85% specificity (95% CI: 99.57%–99.97%), with three samples showing false IR and RR results. In contrast, the Alinity s HIV Ag/Ab Combo assay yielded two false reactive results (specificity: 99.90%; 95% CI: 99.65%–99.99%), while the Alinity i HIV Ag/Ab Combo assay yielded one false RR result (specificity: 99.95%; 95% CI: 99.73%–100%).

**TABLE 1:** Comparison of average specificity among Elecsys assays on the Cobas e 801 analyser and corresponding assays on the Alinity s and Alinity i platforms.

	e 801 analyser	Alinity s	Alinity i
Elecsys HIV Duo and Alinity s/i HIV Ag/Ab Combo (*n* = 2048)	99.85% (99.57%–99.97%)	99.90% (99.65%–99.99%)	99.95% (99.73%–100%)
Elecsys HCV Duo, Elecsys Anti‐HCV II, and Alinity s/i Anti‐HCV (*n* = 2046)	100% (99.82%–100%) 100% (99.82%–100%)	100% (99.82%–100%)	99.80%^a^ (99.50%–99.95)
Elecsys HBsAg II, Alinity s HBsAg, and Alinity i HBsAg Qual II (*n* = 2048)	99.95% (99.73%–100%)	100% (99.82%–100%)	RR: 99.95% (99.73%–100%) IR^b^: 99.80% (99.50%–99.95%)

Abbreviations: Ab, antibody; Ag, antigen; HBsAg, hepatitis B surface antigen; HCV, hepatitis C virus; HIV, human immunodeficiency virus; IR, initially reactive; RR, repeatedly reactive.

^a^ Statistically significant difference in specificity between Alinity i Anti‐HCV and all other HCV assays tested.

^b^ Three samples were IR (before the confirmatory algorithm was applied) on Alinity i HBsAg Qual II.

For the 2046 donor samples tested for HCV, both Elecsys HCV assays demonstrated 100% specificity (95% CI: 99.82%–100%), with no false reactive results. The Alinity s Anti‐HCV II assay also demonstrated 100% specificity (95% CI: 99.82%–100%), indicating the ability of these platforms to detect non‐reactive samples. In contrast, the Alinity i Anti‐HCV assay had four false reactive samples as confirmed by NAT and immunoblotting, yielding 99.80% specificity (95% CI: 99.50%–99.95%). The absolute difference in specificity between the Alinity i Anti‐HCV assay and the other HCV assays evaluated was statistically significant.

The Elecsys HBsAg II assay demonstrated 99.95% specificity (95% CI: 99.73%–100%; *n* = 2048). One sample was consistently RR across all repeat measurements (signal‐to‐cutoff ratio values of 4.70, 4.46 and 4.76), but both NAT and neutralization testing returned negative results, indicating this was a false positive result. The Alinity s HBsAg assay showed 100% specificity (95% CI: 99.82%–100%), with no IR or RR results. The Alinity i HBsAg Qualitative II assay showed 99.95% specificity (95% CI: 99.73%–100%), matching the Elecsys assay's point estimate. Although this assay yielded three IR results, only one sample was RR on retesting, and this was confirmed to be a false positive (Table 1).

### Seroconversion sensitivity

Compared with the Alinity s HIV Ag/Ab Combo assay, the Elecsys HIV Duo assay detected HIV earlier in 8.0% of evaluated seroconversion panels (*n* = 51), in the same time in 84.0% of panels, and later in 8.0% of panels. Similarly, the Elecsys HIV Duo assay showed earlier detection in 8.0% of panels, the same detection time in 86.0% of panels and later detection in 6.0% of panels compared with the Alinity i HIV Ag/Ab Combo assay.

Of the 71 panels initially included for HCV, 10 panels were found not to represent true seroconversion cases and so were excluded. The Elecsys HCV Duo assay showed earlier detection in 87.0% of the 61 evaluated panels, the same detection time in 11.0% of panels and later detection in 2.0% of panels compared with the Alinity s Anti‐HCV II assay. Further, the Elecsys HCV Duo assay showed earlier detection in 91.8% of panels, the same detection time in 6.6% of panels and later detection in 1.6% of panels compared with the Alinity i Anti‐HCV assay.

In contrast, the Elecsys Anti‐HCV II assay detected HCV earlier in 9.8% of 61 evaluated panels, in the same time in 83.6% of panels and later in 6.6% of panels compared with the Alinity s Anti‐HCV II assay. Compared with the Alinity i Anti‐HCV assay, the Elecsys Anti‐HCV II assay showed earlier detection in 23.0% of panels, the same detection time in 75.0% of panels and later detection in 2.0% of panels.

Of the 61 HBsAg panels, one was excluded since all samples tested negative across the three assays, thereby precluding comparison. The Elecsys HBsAg II assay showed earlier detection in 7.0% of 60 evaluated panels, the same detection time in 83.0% of panels and later detection in 10.0% of panels compared with Alinity s HBsAg assay. However, compared with the Alinity i HBsAg Qualitative II assay, the Elecsys HBsAg II assay showed earlier detection in 2.0% of panels, the same detection time in 83.0% and later detection in 15.0% of panels.

**FIGURE 1:**
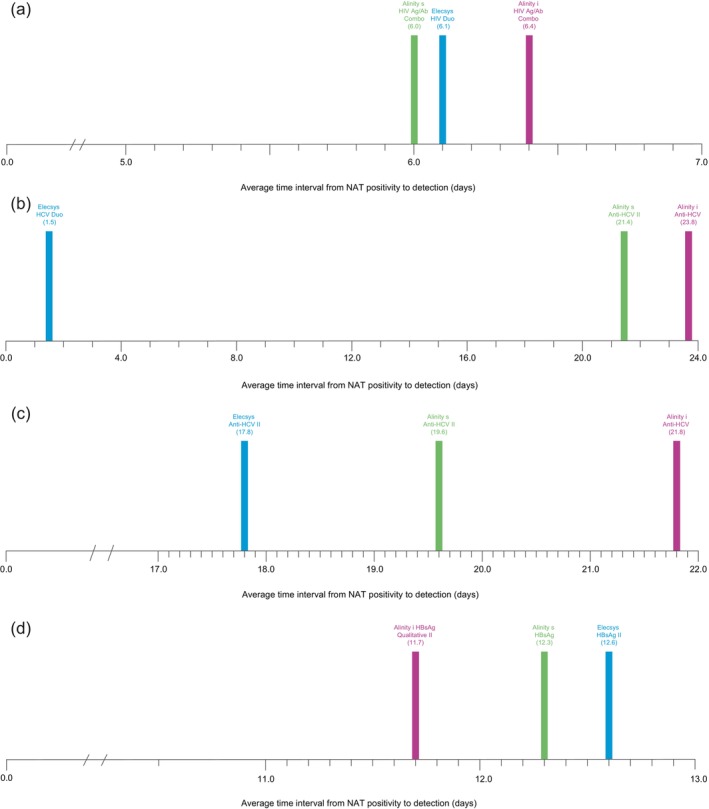
Average time interval from NAT positivity to detection (days). (a) Elecsys HIV Duo assay versus Alinity s and Alinity i HIV Ag/Ab Combo assays (*n* = 49). Day 0 was defined as the day on which the first panel showed positive results for HIV as determined using Chiron HIV‐1 bDNA, Cobas AmpliPrep/Cobas TaqMan® HIV‐1, Cobas Amplicor HIV‐1 Monitor, Gen‐Probe Procleix® HIV‐1/HCV Assay or Roche Ultrasensitive HIV 1 RNA. (b) Elecsys HCV Duo assay versus Alinity s Anti‐HCV II and Alinity i Anti‐HCV assays (*n* = 56). Day 0 was defined as the day on which the first panel showed positive results for HCV RNA as determined using Cobas AmpliPrep/Cobas TaqMan HCV Quantitative Test v2.0, Chiron HCV RNA bDNA 2.0, or ALPHA HCV by PCR/quantitative RNA PCR. (c) Elecsys Anti‐HCV II assay versus Alinity s Anti‐HCV II and Alinity i Anti‐HCV assays (*n* = 27), with the same definition of Day 0 as in 1b. (d) Elecsys HBsAg II assay versus Alinity s HBsAg and Alinity i HBsAg Qualitative II assays (*n* = 60). Day 0 was defined as the day on which the first panel showed positive results for HBV DNA as determined using Cobas AmpliPrep/Cobas TaqMan, Abbott REALTIME HBV DNA m2000®, HBV DNA Roche PCR, or Cobas 6800/8800 HBV). Ab, antibody; Ag, antigen; HBsAg, hepatitis B surface antigen; HCV, hepatitis C virus; HIV, human immunodeficiency virus; NAT, nucleic acid testing.

For the quantitative analysis, the performance of the assays in detecting early infection was examined in subsets of seroconversion panels used in the primary evaluation via a head‐to‐head comparison of the average interval between a positive NAT result and the assay detection time. The Elecsys HIV Duo assay showed an average time to detection of 6.1 days after the first positive NAT test (*n* = 49). The Alinity s HIV Ag/Ab Combo assay had the shortest time to detection, at 6.0 days, while the Alinity i HIV Ag/Ab Combo assay had a slightly longer time of 6.4 days (Figure 1a).

Among the HCV assays evaluated, the Elecsys HCV Duo assay had the shortest average time to detection, identifying seroconversion at 1.5 days after the first HCV RNA‐positive result (*n* = 56). The Alinity s Anti‐HCV II assay detected seroconversion at an average of 21.4 days (19.9 days later than the Elecsys HCV Duo assay), while the Alinity i Anti‐HCV assay showed the longest time to detection of 23.8 days—a 22.3‐day delay relative to the Elecsys HCV Duo assay (Figure 1b).

The Elecsys Anti‐HCV II assay identified anti‐HCV antibody at an average of 17.8 days after the first HCV RNA‐positive result (*n* = 27). The Alinity s Anti‐HCV II assay detected seroconversion 1.8 days later, at an average of 19.6 days. The Alinity i Anti‐HCV assay showed the longest average detection time of 21.8 days—a delay of 4.0 days compared with the Elecsys Anti‐HCV II assay (Figure 1c).

The Elecsys HBsAg II assay detected HBsAg at an average of 12.6 days after NAT positivity (*n* = 60). Detection time was slightly shorter at 12.3 days for the Alinity s HBsAg assay, while the Alinity i HBsAg Qualitative II assay had the shortest average detection time of 11.7 days (Figure 1d).

## DISCUSSION

The present study is the first to provide comprehensive performance data comparing the Elecsys HIV Duo, HCV Duo, Anti‐HCV II and HBsAg II assays with the Alinity s HIV Ag/Ab Combo, Anti‐HCV II and HBsAg assays, as well as with the Alinity i HIV Ag/Ab Combo, Anti‐HCV and HBsAg Qualitative II assays, using samples from first‐time blood donors and commercial seroconversion panels. Head‐to‐head comparative studies such as this one provide critical evidence to support the highest standards of blood safety and ensure efficient implementation of these assays in in vitro diagnostic laboratories.

All four Elecsys assays tested had specificities similar to corresponding Alinity assays. Specificity tends to be lower in first‐time blood donors than in repeat donors, due to the progressive exclusion of false positive and confirmed reactive samples in repeat donor populations by routine assays in use for a longer time [19]. With this in mind, the high specificities of 99.85%–100% observed for all Elecsys assays highlight their performance and confirm their suitability for use in blood donor screening and diagnostic routine testing, where high specificity is critical to avoid unnecessary donor deferral and/or follow‐up testing. The few false reactive results seen with the Elecsys HIV Duo and Elecsys HBsAg II assays were within the expected performance range. Further, the specificities observed for the four Elecsys assays were consistent with those reported in their respective instructions for use, which are based on performance evaluations in multiple centres [20, 21, 22, 23], supporting the robustness of these assays under routine conditions.

With similar times to detection for most seroconversion panels, all four Elecsys assays showed good seroconversion sensitivities. Although the time to detection was slightly later with the Elecsys HBsAg II assay than with corresponding assays on both Alinity platforms, it was within close performance range.

Notably, the Elecsys HCV Duo assay demonstrated earlier detection of HCV infection in approximately 90% of the evaluated seroconversion panels, reinforcing the evidence on potential advantages of using combined antigen and antibody screening assays for HCV instead of antibody‐only screening assays [24]. The Elecsys HCV Duo assay incorporates several design features that may influence analytical sensitivity. These include an alkaline pre‐treatment step intended to disrupt viral particles and release HCV core antigen, as well as buffer and reagent formulations designed to support antigen detection. The assay uses monoclonal antibodies directed against conserved regions of the HCV core protein, enabling detection across HCV genotypes. In addition, antigen and antibody components are processed in parallel rather than within a combined reaction, allowing each to be optimized independently [25]. These methodological characteristics may contribute to the assay's ability to detect HCV antigen early in infection, with important implications for diagnostic use as well as for blood donor screening in countries that have not routinely adopted NAT [24].

When benchmarked against the first NAT‐positive result, the performances of the Elecsys HIV Duo and Elecsys Anti‐HCV II antibody assays were similar to those of corresponding Alinity s and Alinity i assays: HIV seroconversion was detected approximately 6 days after NAT positivity and HCV at approximately 17–22 days. Notably, the Elecsys Anti‐HCV II assay detected HCV antibody at a shorter interval post‐NAT positivity than the Alinity s Anti‐HCV II and Alinity i Anti‐HCV assays. The interval between HCV NAT positivity and seroconversion with the Elecsys HCV Duo assay was even shorter, at just 1.5 days, representing a substantial improvement in early HCV detection and confirming its ability to narrow the serological window towards the infection time point.

The study has several strengths. Many studies in blood screening use regular repeat donors, which introduces preselection bias in specificity analyses. When comparing screening assays using samples from regular repeat donors, newer tests tend to be less specific than those in long‐term routine use. This is because, based on the findings of older tests, samples from donors with confirmed or false positive results are excluded, making the donor pool more selective [19, 26, 27, 28]. This bias was circumvented in the present study by using samples from first‐time blood donors. Then, sensitivity comparisons are hampered by use of small numbers of archived, difficult‐to‐characterize positive samples. The large number of seroconversion panels used in the sensitivity analysis enabled comprehensive comparison of early detection capabilities across the e 801 analyser and Alinity s and Alinity i platforms. Conventional sensitivity studies use isolated samples, which may mask nuanced differences in early detection performance. The use of seroconversion panels in the present study facilitated identification of these differences. Using the first NAT‐positive result as a benchmark, the sensitivity analysis also compared how early each assay could detect infection. This approach offers a more meaningful assessment of sensitivity than traditional methods that rely on single, predefined specimens from various disease stages, which often yield uniformly high sensitivity values (e.g., 100%) across all assays. The inclusion of a quantitative metric in the seroconversion sensitivity analysis (average number of days between the first NAT‐positive result and assay reactivity) further characterized the ability of each assay to detect infections in the earliest phase, before full antibody development.

Notwithstanding these strengths, certain limitations should be noted. The time intervals between sample draws and total number of available bleeds varied among the seroconversion panels evaluated, which could have impacted the precision of time‐to‐detection estimates. Second, infectious disease assays used for screening and in vitro diagnostics must demonstrate high seroconversion sensitivity across genotypes (HIV or HCV) and mutants (HBV), and previous studies have shown that Elecsys assays perform well across diverse genotypes and mutants [29, 30, 31]. However, currently available seroconversion panels are exclusively from the United States and therefore represent only the most prevalent genotypes in infected individuals from that region (e.g., genotype B for HIV). Thus, the early infection and seroconversion data results presented here are limited to a narrow range of genotypes, leaving others underrepresented. Future studies should expand the evidence base for the four Elecsys assays evaluated by including well‐characterized specimens from diverse geographical regions when these become available.

In conclusion, the findings of the present study, bolstered by data from a large and diverse set of seroconversion panels and an unbiased cohort of first‐time donors, support routine use of Elecsys assays in blood donor screening for safeguarding blood supplies and facilitating early diagnosis of transfusion‐transmissible infections.

## CONFLICT OF INTEREST STATEMENT

F.P., M.C. and M.S. are employees of Roche, the sponsor of this study. F.P. and M.C. hold stocks/stock options with Roche.

## Supporting information


**Data S1.** Supporting information.

## Data Availability

Reasonable requests for datasets generated during and/or analysed during the current study may be directed to rotkreuz.datasharingrequests@roche.com.
