# Supplementary material for: Comparing specificity and seroconversion sensitivity among major blood screening assays for human immunodeficiency virus and viral hepatitis
Source: Vox Sang. 2026 Feb 17;121(6):814–21. doi: 10.1111/vox.70222 (PMC13253053; doi:10.1111/vox.70222)
Supplement: Supplementary file 1 — Data S1. Supporting information. [file VOX-121-814-s001.docx]

**Supplementary methods**

Confirmatory algorithm for discrepancies between Elecsys^®^ and Abbott Alinity s/i assays

If a result was discrepant between the Elecsys assays and the corresponding Alinity s/i assays, the following confirmatory algorithm was used for resolution: For HIV, polymerase chain reaction (PCR) was used for positive antigen module results, and immunoblot (Fujirebio) was used for positive antibody module results. For HCV, immunoblot (Fujirebio) was used for anti-hepatitis C virus (HCV)-positive results. PCR was used for positive antigen module results, while immunoblot (Fujirebio) was used for positive antibody module results. Lastly, hepatitis B surface antigen (HBsAg) neutralisation and nucleic acid testing were used for HBsAg-positive results (HbsAg confirmatory assay).

Table S1. Interpretation of results from Elecsys assays on the Cobas^®^ e 801 analyser

| **Assay** | **Cut-off index** | **Interpretation** |
| --- | --- | --- |
| Elecsys HIV Duo | <1.0  ≥1.0 | Non-reactive  Reactive |
| Elecsys HCV Duo | <1.0  ≥1.0 | Non-reactive  Reactive |
| Elecsys Anti-HCV II | <0.9  ≥0.9 to <1.0  ≥1.0 | Non-reactive  Borderline  Reactive |
| Elecsys HBsAg II | <0.9  ≥0.9 to <1.0  ≥1.0 | Non-reactive  Borderline  Reactive |

Table S2. Interpretation of results from Alinity s assays

| **Assay** | **Cut-off index** | **Interpretation** |
| --- | --- | --- |
| Alinity s HIV Ag/Ab Combo | <1.0  ≥1.0 | Non-reactive  Reactive |
| Alinity s Anti-HCV II | <1.0  ≥1.0 | Non-reactive  Reactive |
| Alinity s HBsAg | <1.0  ≥1.0 | Non-reactive  Reactive |

Table S3. Interpretation of results from Alinity i assays

| **Assay** | **Cut-off index** | **Interpretation** |
| --- | --- | --- |
| Alinity i HIV Ag/Ab Combo | <1.0  ≥1.0 | Non-reactive  Reactive |
| Alinity i Anti-HCV | <1.0  ≥1.0 | Non-reactive  Reactive |
| Alinity i HBsAg Qualitative II | <1.0  ≥1.0 | Non-reactive  Reactive |
